# Surgical Outcomes of Displaced Metaphyseal Distal Radius Fractures in Children: A Consecutive Case Series of 100 Patients

**DOI:** 10.7759/cureus.107927

**Published:** 2026-04-28

**Authors:** Qamar Mustafa, Barry Mullins, Harold Akehurst, Darren Roberts

**Affiliations:** 1 Trauma and Orthopaedics, Southampton General Hospital, University Hospital Southampton NHS Foundation Trust, Southampton, GBR; 2 Trauma and Orthopaedics, Queen Alexandra Hospital, Portsmouth Hospitals University NHS Trust, Portsmouth, GBR; 3 Trauma and Orthopaedics, Bristol Royal Hospital for Children, Bristol, GBR

**Keywords:** bone remodelling, kirschner wire, k-wire, manipulation under anaesthesia, mua, paediatric distal radius fracture, paediatric orthopedics

## Abstract

Background: Distal radius fractures are among the most common type of fracture seen in the paediatric population. Manipulation under anaesthesia (MUA), with or without Kirschner wire (K-wire) fixation, is the standard treatment for metaphyseal fractures. This study assessed outcomes by fracture type and operative modality.

Methods: One hundred consecutively surgically treated metaphyseal distal radius fractures in children aged 4-10 years were retrospectively reviewed in our centre. Treatments included MUA with cast alone (MUA/cast) or MUA with K-wire fixation (MUA/K-wire). Fracture type, complications, return to theatre, and time to union/discharge were analysed.

Results: Of 100 patients (46 female, 54 male; mean age 7.4 years), 59 had angulated fractures and 41 had off-ended fractures. 44 (75%) angulated fractures were treated with MUA/cast, while 33 (80%) off-ended fractures were managed with MUA/K-wire. Seven (7%) patients required further surgery for redisplacement. In the off-ended group, redisplacement occurred in two of eight (25%) MUA/cast cases compared with 2 of 33 (6%) MUA/K-wire cases (p=0.16). Overall, K-wire augmentation showed a trend towards reduced risk of re-operation (p=0.059). All fractures united (mean 44.5 days); however, off-ended fractures took significantly longer to heal (p=0.04). Among 48 MUA/K-wire cases, 15 (31%) developed complications, including wire migration, irritation, granulation tissue, hypertrophic scarring, and return for wire removal. Seven malunions remodelled spontaneously, including one from 25° dorsal displacement to normal alignment.

Conclusion: Off-ended fractures treated with MUA/cast were more likely to redisplace than those treated with MUA/K-wire, though K-wire fixation carried higher complication rates. Given the excellent remodelling potential in children aged 4-10 years, MUA/cast is recommended where stability can be achieved, as it reduces complications while still allowing reliable remodelling.

## Introduction

Introduction

Distal radius fractures are the most common fracture accounting for up to 30% of all injuries within the paediatric population [1-3]. Metaphyseal distal radius fractures are defined as fractures within 4 cm proximal to the distal radius physis [4]. These fractures may be isolated to the radius (AO 23r-M/2.1/3), involve the ulna as a both bone forearm fracture (AO 23-M2.1/3) [5]. A greenstick fracture, a combination of a complete fracture on the tension side and plastic deformity on the compression side, and torus fractures, where a single cortex has failed in compression may not require any manipulation. Displaced fractures with unacceptable visual angulation or complete discontinuity of the cortex (off-ended) usually require orthopaedic intervention [6,7].

In the United Kingdom (UK), distal radius fractures in children are more likely to be treated by manipulation with possible augmentation using Kirschner wires (K-wires) compared to other countries [8]. Currently, there is no consensus on the correct treatment of these injuries. There is evidence to suggest that these fractures will unite and remodel to anatomically acceptable alignment without intervention in children under 11 years old; however, the quality of evidence is poor [9,10]. In the upper extremity, longitudinal growth in the physis of the distal radius and ulna, confers considerable modelling potential.

Currently displaced metaphyseal distal radius fractures are treated with closed reduction and immobilisation in a plaster cast using sedation in the emergency department or under general anaesthetic in the operating theatre. In the upper extremity, longitudinal growth in the physis of the distal radius and ulna confers considerable modelling potential. Therefore, manipulation may not be required if the fracture is in a stable position [11]. Noonan and Price suggested a fracture was unstable and recommended intervention if fracture displacement exceeded >15 degrees angulation, >45 degrees rotation or if a bayonet deformity was present [6]. K-wire fixation of distal radius fractures has been recommended when closed reduction has failed and the fracture pattern is unstable [7]. However, K-wire fixation is associated with complications including: pain, pin site infection, loss of reduction, scarring, neuropraxia or further anaesthesia to remove the wires [11-13].

There is a clear need to inform patients of the risks and benefits of operative and non-operative treatment for these injuries. Indeed, the British Society of Children's Orthopaedic Surgery has recently prioritised the management of distal radius fractures as one of their most important research questions [14].

The aim of this retrospective study is to identify differences in outcome depending on fracture type and operative modality of displaced metaphyseal distal radius fractures treated with either manipulation under anaesthetia and cast (MUA/cast) or manipulation under anaesthetia and K-wire fixation (MUA/K-wire). We investigated time to union both radiographically and clinically, rate of return to theatre and the incidence of complications based on initial fracture pattern and treatment modality.

## Materials and methods

Study design****

The study was conducted at the Department of Trauma and Orthopaedics of Queen Alexandra Hospital, located in Portsmouth, UK. This was designed as a retrospective review of 100 consecutive fractures in children aged 4-10 years old who underwent MUA with casting (MUA/cast) or MUA and Kirschner wiring (MUA/K-wire) for severely displaced metaphyseal distal radius fractures. Data was collected retrospectively from the department's trauma database for consecutive cases between 2015-2019 and determined by the number of available cases available. Prior to commencement, the project was registered with the hospitals clinical audit department, in accordance with local governance procedures. 

Study population

Inclusion criteria included children aged 4-10 years, with radiological evidence of a severely displaced metaphyseal radius fracture. Severely displaced was defined as an off-ended fracture or angulated fracture as determined by Noonan and Price [6]. Metaphyseal was defined as within 4 cm of the physis. Exclusion criteria included children with multiple fractures, closed growth plates, open fractures or patients who did not complete at least four weeks of follow-up were excluded. Due to the database used, children that had manipulation under sedation in the emergency department were not analysed within this cohort.

Surgical procedure

All procedures consisted of a standard pre-operative management with attention to analgesia, neurovascular assessment and initial stabilisation in above elbow back-slab plaster cast, in a position of comfort. Following review at trauma meeting, a provisional management plan was made but definitive surgical decision was made intra-operatively by the responsible consultant based on fracture stability and alignment following manipulation. Where K-wire was used, AO technique was utilised using a a single, two or three crossed smooth 1.6mm wires through the radial metaphysis, avoiding the growth plate and perichondral ring [5]. K-wires were left protruding through the skin, bent, cut and protected with sterile padding prior to application of a short arm cast. All cases were performed under general anaesthesia and image intensifier control in the operating theatre and all children were treated by orthopaedic surgeons with experience in paediatric trauma surgery. Follow up was initially at 7-10 days post-operatively. Further follow up was determined by time to union, determined radiologically, with removal of K-wires done in an outpatient setting. 

Study measures

Fracture characteristics, treatment modality, return to theatre, complications and time to union/discharge were analysed. 

Statistical analysis

The data collected was entered in a Microsoft Excel (Microsoft Corp., USA) document. Time to union was evaluated using Kaplan-Meier curves and log-rank tests. A descriptive analysis of demographics, fracture characteristics and treatments were undertaken and represented in tables. Associations between fracture or treatment characteristics and return to theatre or complication rates were assessed using Fisher’s exact test. Statistical significance was accepted if p- value ≤ 0.05. Statistical analysis was performed in R version 4.2.2 (R Core Team, Vienna).

## Results

Demographics and injuries

100 operatively treated metaphyseal distal radius fractures were reviewed. There were 46 female and 54 male patients aged between 4 and 10 years. The mean age was 7.4 years old.

There were 53 left and 47 right-sided fractures. The ulna was fractured in 72 cases. None of the ulna fractures required K-wire fixation. Deformity is summarised in Table 1. 41 fractures were off-ended (41%). Of the remaining 59 fractures with preserved cortical contact, the majority were dorsally displaced with a mean angulation of 28.1° (88%). Fractures were more likely to be radially deviated rather than neutral or ulnar deviated, with a mean deformity 12.4°.

**Table 1:** Classification of fractures by deformity * Deformities are described only for fractures with preserved bony contact.

Fractured Characteristic	Deformity Type	Frequency n (%)	Mean Angulation (°) ± SD
Bony contact (n = 100)	Angulated	59	
	Off-ended	41	
Sagittal deformity (n = 58)*	Volar	7 (12%)	17.0 ± 3.6°
	Dorsal	51 (88%)	28.1 ± 9.6°
Coronal deformity (n = 58)*	Neutral	13 (22%)	0.5 ± 1.5°
	Radial	39 (67%)	12.4 ± 8.0°
	Ulnar	6 (10%)	12.9 ± 7.3°

Treatment

Operative treatment was planned primarily for 87 patients and the remaining 13 were treated because of loss of satisfactory initial alignment; in one case, this was due to subsequent further trauma. Mean time to theatre in cases planned primarily was 0.8 ± 1.2 days, and 13.2 ± 6.2 days for those planned following loss of alignment. Planned follow up was performed at weekly intervals to monitor loss of alignment. 

As per surgeon preference, one patient received MUA and below elbow cast, whereas 51 patients received MUA and above elbow cast; 48 patients received MUA and K-wire fixation. A single wire was used in 24 cases, two wires were used in 23 cases, and three wires were used in one case, again subject to surgeon preference.

Off-ended fractures were more likely to be treated with MUA/K-wire compared to angulated fractures (33, 80% vs 15, 25%) and were also more likely to receive more than one K-wire (19, 57% vs 5, 33%) (Table 2). 

**Table 2:** Treatment by fracture type K-wire: Kirschner wire

Treatment Intervention		Bony Contact	
		Angulated, n (%)	Off-ended, n (%)
MUA + below elbow cast		1 (1%)	0 (8%)
MUA + above elbow cast		43 (73%)	8 (20%)
MUA + K-wire fixation		15 (25%)	33 (80%)
1 K-wire		10 (67%)	14 (42%)
2 K-wires		5 (33%)	18 (54%)
3 K-wires		0 (0%)	1 (3%)

Union

**Figure 1:**
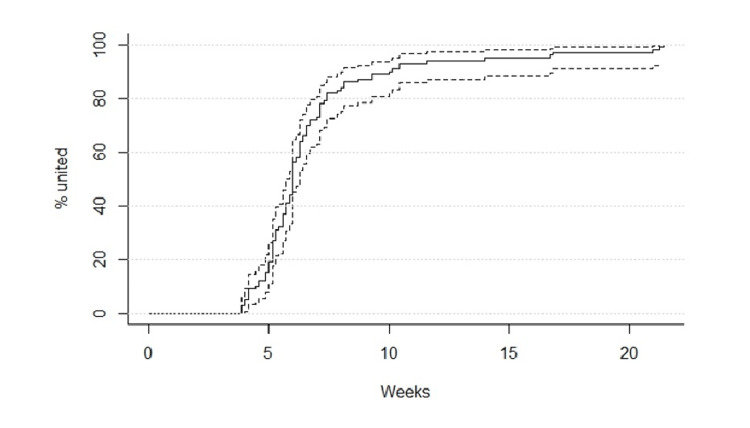
Graph showing time to union with median and IQRs IQR: Interquartile range

All fractures went on to union with a median time to union of 44.5 days (range 27 - 150 days; interquartile range (IQR) 14 days) (Figure 1).

Although median time to union was similar for off-ended and angulated fractures (43 and 41 days, respectively), there were several long outliers in the off-ended group due to infection, including one case of osteomyelitis, following closed reduction and K-wire fixation. This is likely due to infection delaying union and these patients requiring further monitoring as a result. This did not reach the threshold for statistical significance for overall time to union (p>0.05), in the off-ended group versus preserved contact group.

**Figure 2:**
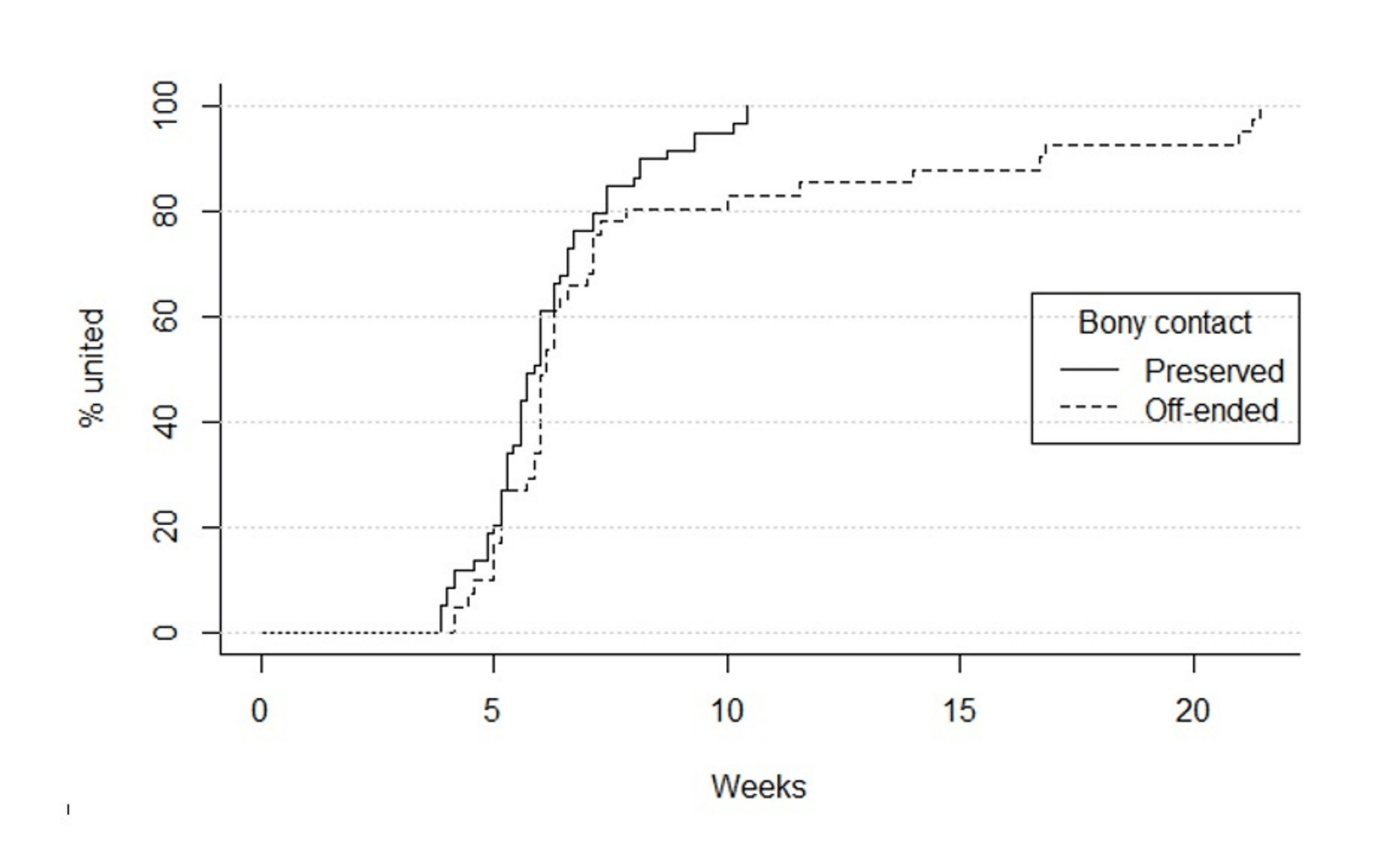
Graph showing time to union depending on initial bony contact (preserved vs off-ended)

There was no relationship between initial procedure and time to union either across the whole cohort (p = 0.20) or specifically among off-ended fractures (p = 0.40). Though off-ended fractures took longer to achieve union than fractures where bony contact was preserved, at time of initial presentation, all fractures went on to achieve union (Figure 2). 

Seven instances of marked remodelling of malunion were observed, regardless of initial bony contact with one fracture remodelling from 25° dorsal displacement to normal alignment. All patients were discharged between six and 28 weeks following the date of injury, with no further re-admissions for delayed complications (Figure 2). 

Return to theatre

Due to redisplacement of the fracture seven patients returned to theatre for a second procedure (7%) (Tables 3, 4, 5). One of these was further complicated by osteomyelitis secondary to K-wire infection. Three were angulated fractures and four were off-ended. If originally treated with MUA/cast the fracture was then augmented with at least one K-wire. If a single K-wire was already in situ then the number of wires were increased, or in one case, open reduction internal fixation (ORIF) was performed. 

**Table 3:** Characteristics of patients requiring surgical re-intervention (n=7), including initial management, specific hardware used, subsequent procedure, and time elapsed between operations. MUA: Manipulation under anaesthesia; POP: Plaster of Paris; K-wire: Kirschner wire; ORIF: Open reduction internal fixation

First procedure	Wires	Second Procedure	Interval (Days)
MUA/POP above elbow	0	MUA + 1 K-wire	6
MUA/POP above elbow	0	MUA + 1 K-wire	11
MUA/POP above elbow	0	MUA + 1 K-wire	15
MUA/POP above elbow	0	MUA + 2 K-wires	11
MUA + K-wire fixation	1	MUA + 2 K-wires	9
MUA + K-wire fixation	1	MUA + 3 K-wires	9
MUA + K-wire fixation	1	ORIF	27

**Table 4:** Complication rates according to initial fracture presentation - angulated vs off-ended. No statistical significance was found for the different complications. * Fisher’s exact test K-wire: Kirschner wire

Complication	Total	Bony Contact		p-value*
		Angulated (n = 59)	Off-ended (n = 41)	
Loss of reduction	7	3 (5%)	4 (10%)	0.44
Ulnar nerve palsy	1	1 (2%)	0 (0%)	1.00
K-wire migration	4	2 (3%)	2 (5%)	1.00
K-wire site infection	2	0 (0%)	2 (5%)	0.17
K-wire site problem (not infected)	8	3 (5%)	5 (12%)	0.27

**Table 5:** Complication rates categorised by primary treatment method (MUA/cast vs. MUA/K-wire) and by the number of K-wires used K-wire: Kirschner wire; MUA: Manipulation under anaesthesia

Complication	Treatment				Number of Wires			
	MUA/cast (n = 52)	MUA/K-wire (n = 48)	p-value*	1 wire (n = 24)	2 wires (n = 23)	3 wires (n = 1)	p-value*	
Loss of reduction	4 (8%)	3 (6%)	0.44	3 (13%)	0 (0%)	0 (0%)	0.28	
Ulnar nerve palsy	1 (2%)	0 (0%)	1.00	0 (0%)	0 (0%)	0	1.00	
K-wire site problem (not infected)		4 (8%)		3 (13%)	1 (4%)	0	0.64	
K-wire site infection		2 (4%)		2 (8%)	0 (0%)	0	0.51	
K-wire migration		7 (15%)		3 (13%)	4 (17%)	0	0.74	

**Figure 3:**
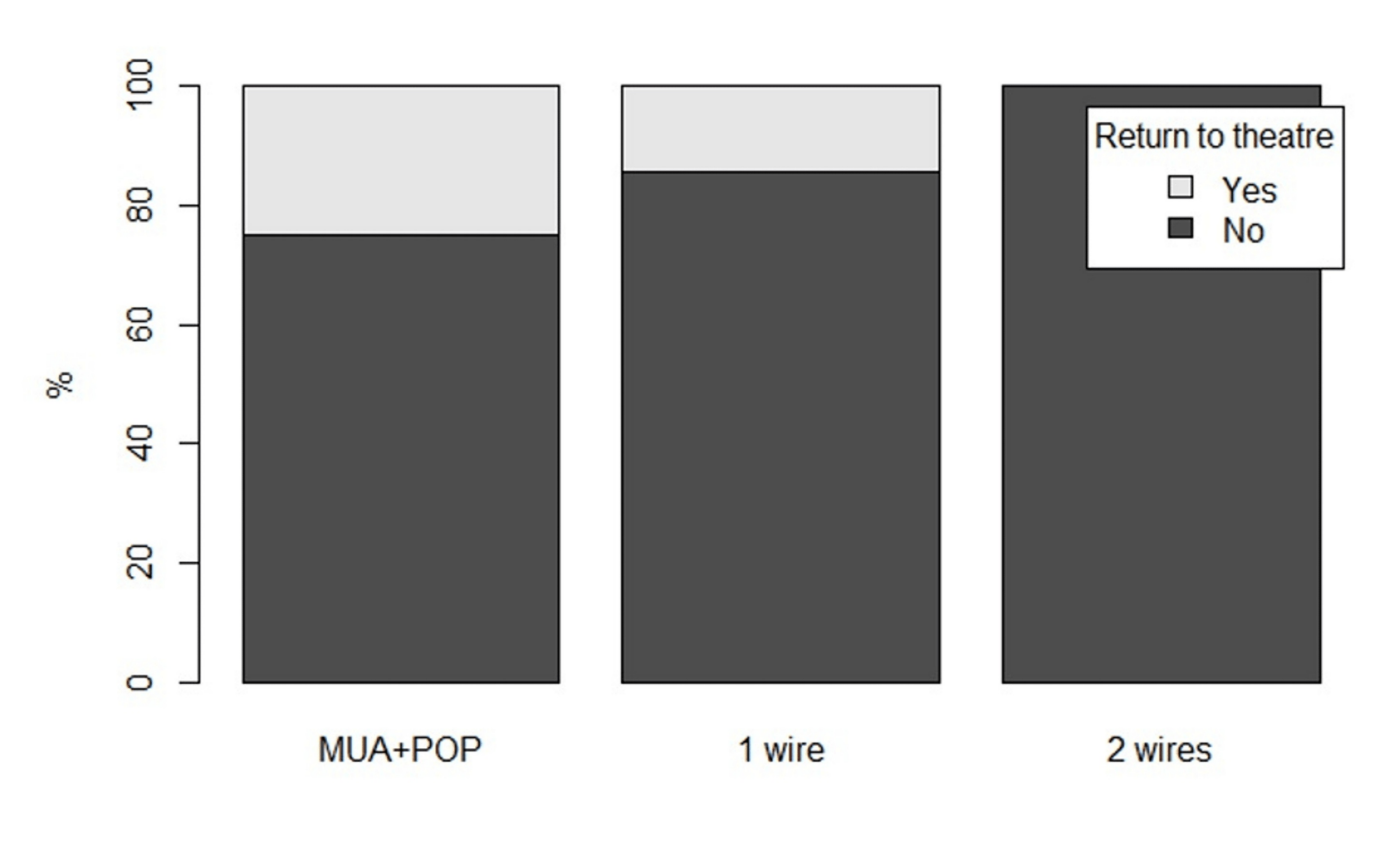
Return to theatre rate in offended fractures expressed as a percentage depending on type of initial stabilisation method MUA: Manipulation under anaesthesia; POP: Plaster of Paris

Within the off-ended group a higher proportion of those treated with cast compared to K-wire augmentation underwent a second procedure (p=0.16), this equating to two of eight MUA/cast (25%) vs two of 33 MUA/K-wire (6%). Comparing cast alone versus number of K-wires, there was a trend towards decreasing risk of return to theatre with supplementary K-wires (p=0.059) (Figure 3). Further two patients required return to theatre for removal of K-wires under general anaesthetic. 

Complications

Apart from loss of reduction, 15 of the 48 MUA/K-wire procedures had additional complications (31%). These included K-wire migration or irritation, granulation tissue, hypertrophic scar and return to theatre for K-wire removal (Table 4). There were two instances of infection, including once instance of osteomyelitis which required antibiotics, delayed union and time to union.

One individual who was treated with MUA and above elbow cast sustained an ulnar nerve palsy, which subsequently fully recovered. There was no correlation between type of fracture and type of complication, nor any correlation between number of K-wires and type of complication (Table 5).

All patients were discharged between six and 28 weeks following the date of injury, with no further re-admissions for delayed complications. 

## Discussion

The management of displaced metaphyseal distal radius fractures in children remains a topic of considerable debate. Closed reduction and cast immobilisation has long been the standard of care but concerns regarding loss of reduction have led many surgeons to favour supplementary K-wire fixation, particularly for unstable patterns. Although this has been shown to give functional results in most cases, loss of reduction in cast is a well-documented complication, which may result in a poorer outcome if associated with malunion [4,7,9]. Our series highlights the clinical trade-offs between these two approaches.

With respect to metaphyseal fractures, further displacement is common (approximately 35% over all paediatric ages) and K-wire augmentation is often utilised to reduce this risk [15-21]. Therefore, MUA and K-wire fixation is more likely with significant initial displacement as seen with off-ended fractures [18]. This practice was observed in our study with 75% of angulated fractures being treated with MUA/cast in contrast to 80% off-ended fractures managed with MUA/K-wire.

The overall risk of redisplacement requiring further surgical intervention following initial operative management is 15%. The rate of requiring a second operation after further displacement was similar with respect to angulated fractures and off-ended fractures. Likewise, similar rates were observed comparing all cases of MUA/cast and MUA/K-wire. However, off-ended distal radial fractures treated with MUA/cast were more likely to displace again to the point of requiring further surgical intervention when compared to fractures that underwent K-wire fixation, although this did not reach statistical significance. Cast moulding, measured through cast index and gap index, has been noted to be a factor in the successful maintenance of reduction [11]]. A limitation in this study is that quality of the casting technique, including cast index, could not be identified to determine whether the variation in cast immobilisation impacted on rate of displacement.

Within our cohort, a 31% complication rate was observed in those who were treated with K-wire fixation. An additional anaesthetic was required in two of these children to remove the K-wires. Miller et al. randomised 34 children with metaphyseal distal radius fractures and among the K-wire group; there was a 38% pin-related complication rate [4]. McLauchlan et al. randomised 68 children to either closed reduction and cast immobilisation versus immediate K-wire fixation [13]. Loss of reduction was seen in 21% and 0% of patients treated with casting versus pinning, respectively. Despite a 6% rate of pin-related complications, clinical function at three months after injury did not significantly differ between groups.

We had one case of deep infection (osteomyelitis), which was successfully treated with long term antibiotics and monitoring of the fracture to union. Subacute osteomyelitis following K-wire insertion in distal radius fractures is very rare and single deep infections are reported as 0.76% in open reduction and pin fixation [22-24]. In most cases, complications will be resolved by removing the pin without any long-term problems [23].

Given this was a retrospective study, clinic outcomes including patient satisfaction were not assessed. However, all patients were discharged by a maximum of seven months following clinical and radiological union, without ongoing sequelae of these complications. Nevertheless, it is paramount the children and their relatives must be counselled regarding these potential risks that can occur in nearly one third of cases treated with K-wires.

Distal radius fracture treatment remains an area of controversy in the paediatric population. The findings of remodelling in this study corroborate other work which suggests that metaphyseal distal radius fractures are an area of clinical equipoise. Seven instances of marked remodelling of malunion were observed, with one fracture remodelling from 25° dorsal displacement to normal alignment. In Do et al.'s study, the radius was allowed to heal in a shortened position after redisplacement with no reported complications at the time of final follow up [12].

We advocate MUA/cast as the preferred treatment for both angulated and off-ended metaphyseal distal radius fractures in 4-10 year olds, provided stability is achieved. K-wires should be reserved for unstable fractures where reduction cannot be maintained in a cast. These conclusions reinforce the need for high-quality prospective data, such as the ongoing Children’s Radius Acute Fracture Trial (CRAFFT), to guide definitive management strategies.

## Conclusions

Both MUA/cast and MUA/K-wire fixation represent techniques which are used to treat displaced metaphyseal distal radius fractures in 4-10 year olds. Off-ended fractures treated in cast have a higher rate of displacement that require a secondary procedure. However, K-wires are associated with higher complications. Remodelling potential is high in this age group. If initial stability can be achieved, we recommend MUA/cast for paediatric angulated and off-ended metaphyseal distal radius fractures, given the reduction in complication rates and high likelihood of remodelling.
